# Prognostic differences in grading and metastatic lymph node pattern in patients with small bowel neuroendocrine tumors

**DOI:** 10.1007/s00423-023-02956-8

**Published:** 2023-06-19

**Authors:** Lisa Reinhard, Martina T. Mogl, Fabian Benz, Agata Dukaczewska, Frederike Butz, Eva Maria Dobrindt, Frank Tacke, Johann Pratschke, Peter E. Goretzki, Henning Jann

**Affiliations:** 1 Department of Surgery, Campus Charité Mitte | Campus Virchow-Klinikum, Charité – Universitätsmedizin Berlin, Corporate Member of Freie Universität Berlin, Humboldt-Universität Zu Berlin, and Berlin Institute of Health, Berlin, Germany; 2 Department of Hepatology and Gastroenterology, Campus Charité Mitte | Campus Virchow-Klinikum, Charité – Universitätsmedizin Berlin, Corporate Member of Freie Universität Berlin, Humboldt-Universität Zu Berlin, and Berlin Institute of Health, Berlin, Germany

**Keywords:** Si-NET, GEP-NET, Grading, Lymph node metastases

## Abstract

**Purpose:**

Neuroendocrine tumors of the small intestine (si-NET) describe a heterogenous group of neoplasms. Based on the Ki67 proliferation index si-NET are divided into G1 (Ki67 < 2%), G2 (Ki67 3–20%) and rarely G3 (Ki67 > 20%) tumors. However, few studies evaluate the impact of tumor grading on prognosis in si-NET. Moreover, si-NET can form distinct lymphatic spread patterns to the mesenteric root, aortocaval lymph nodes, and distant organs. This study aims to identify prognostic factors within the lymphatic spread patterns and grading.

**Methods:**

Demographic, pathological, and surgical data of 208 (90 male, 118 female) individuals with si-NETs treated at Charité University Medicine Berlin between 2010 and 2020 were analyzed retrospectively.

**Results:**

A total of 113 (54.5%) specimens were defined as G1 and 93 (44.7%) as G2 tumors. Interestingly, splitting the G2 group in two subgroups: G2 low (Ki67 3–9%) and G2 high (Ki67 10–20%), displayed significant differences in overall survival (OS) (*p* = 0.008) and progression free survival (PFS) (*p* = 0.004) between these subgroups. Remission after surgery was less often achieved in patients with higher Ki67 index (> 10%). Lymph node metastases (N +) were present in 174 (83.6%) patients. Patients with isolated locoregional disease showed better PFS and OS in comparison to patients with additional aortocaval and distant lymph node metastases.

**Conclusion:**

Lymphatic spread pattern influences patient outcome. In G2 tumors, low and high grading shows heterogenous outcome in OS and PFS. Differentiation within this group might impact follow-up, adjuvant treatment, and surgical strategy.

## Introduction

Neuroendocrine tumors (NET) describe a rare, heterogenous group of malignant neoplasms with significantly rising, but still relatively low incidence varying between 0.32/100,000 and 6.89/100,000 individuals per year across all tumor sites [1–6]. About 70% of all NET occur in the gastroenteropancreatic system (GEP), small intestinal neuroendocrine tumors (si-NET) being the most common. [2, 6–9].

Si-NET, graded almost exclusively into G1 and G2 tumors, often present as small primary tumors with large local and regional lymph node metastases as well as distant organ metastases [8, 10, 11]. These distinct patterns of si-NET metastatic pathways may also influence patient prognosis significantly [10, 12]. In the present study, we, therefore, analyzed the impact of various prognostic factors in si-NET on the prognosis of affected patients, with a focus on tumor grading and the different patterns of lymphatic spread.

As si-NET encompass indolent and slowly growing to highly aggressive tumors, exact prognostic stratification remains challenging [8, 13]. Reliable predictive parameters beside the Ki67 cell proliferation index have yet to be evaluated. In a recent retrospective study, Merola et al. introduced a risk score based on proliferative index, resection status (R), and disease extent for G1/G2 GEP NET to predict the risk of recurrence in radically resected patients [14]. Further, Evers et al. characterized factors associated with recurrence in curatively resected locoregional si-NET such as age, tumor size, and lymph node ratio [15].

The grading system for midgut NET, based on Ki67-index or mitotic count (MC), was established according to grading in foregut NET in 2007 [16]. The proliferation fraction is the most influential independent prognostic factor throughout all NET [13, 16, 17]. Cut-off values for Ki67-index were discussed for certain GEP-NET entities [18], but there are only few studies evaluating the cut-off values for si-NET [19, 20].

Over the years increasing attention was paid to lymph node (LN) involvement. In contrast to the primary tumor being relatively small, si-NET tend to develop early and large mesenteric LN metastases [21] and can also result in mesenteric fibrosis, angulation and ischemia, which often lead to diagnosis of the tumor during emergency operations [10, 22].

European Neuroendocrine Tumor Society (ENETS) guidelines classify LN status as N0 or N1 depending on absence or presence of LN metastases [13, 23]. However, recent tumor, nodes, metastases (TNM) classification differentiates LN metastasis for si-NET into N0, N1 (up to 12 LN) and N2 (more than 12 LN, or bulks of LN in the mesenterium) [24]. The prognostic significance of the number of resected LN as well as the lymphatic spread to the mesenterium is broadly discussed [10].

Controversial discussion about adequate treatment and surgical resection regarding tumor stage is ongoing. Radical surgical resection with sufficient lymphadenectomy (> 8 LN) is recommended for all localized si-NET. In symptomatic patients with advanced NET radical or palliative resection is recommended, if possible. But it is questionable as a pure prophylactic treatment in all asymptomatic patients with advanced NET to prevent future complications [23].

The present retrospective study of 208 patients with si-NET aims to evaluate the significance of metastatic LN patterns for recurrence-free and overall survival (OS) and the heterogenic behavior of G2 tumors within the current grading system. Further, negative prognostic markers for recurrence-free survival and OS are assessed.

## Material and methods

The database of Charité Comprehensive Cancer Centre (CCCC) was searched for patients (age > 18 years) with sporadic small bowel NET (G1 and G2 si-NET) as defined by World Health Organisation (WHO) treated at the ENETS Centre of Excellence at the Charité University Medicine in Berlin, Germany, between January 2010 and December 2020. Patients’ epidemiological data including sex, age, date of diagnosis, and, if applicable, date of death, was transferred in anonymous form to a common database. Exclusion criteria were the presence of genetic syndromes (i.e., von Hippel–Lindau syndrome, multiple endocrine neoplasia type 1), primary tumor site other than the small bowel and G3 histology.

Patient charts along with tumor board protocols were reviewed retrospectively for initial diagnostics, cross-sectional imaging and presence of hormonal hypersecretion syndrome. Surgical reports were examined for type and number of surgeries performed, surgical indication, and intraoperative findings. Data about two-stage or simultaneous liver resection, resection of additional organs, extension of lymphadenectomy and palliative debulking procedures were recorded.

In suspicion of si-NET preoperative assessment included blood tests (standard blood test, chromogranin A (CgA), staging imaging (computed tomography (CT)), magnetic resonance imaging (MRI), and/or somatostatin-receptor imaging and/or DOTATOC-PET-CT (DOTA(0)-Phe(1)-Tyr(3))octreotide—positron emission tomography) and, if applicable, histopathological sampling through endoscopy and/or biopsy of metastases. In an emergency setting (i.e. bowel obstruction, ischemia), diagnostics included a standard emergency blood test (blood cell count, inflammatory values, kidney function, electrolytes, etc.) and radiological examination by CT.

Tumor grading determined by Ki67 proliferation index and MC, TNM classification, multifocality and tumor-specific immunohistochemical staining for Synaptophysin and CgA were gathered from pathology reports of resected specimens. Specification about lymphovascular, perineural and vascular invasion was recorded if assessed in pathology reports. If necessary, histopathological diagnosis was corrected and adjusted regarding the 8th edition of the UICC classification of malignant tumors [24].

**Fig. 1:**
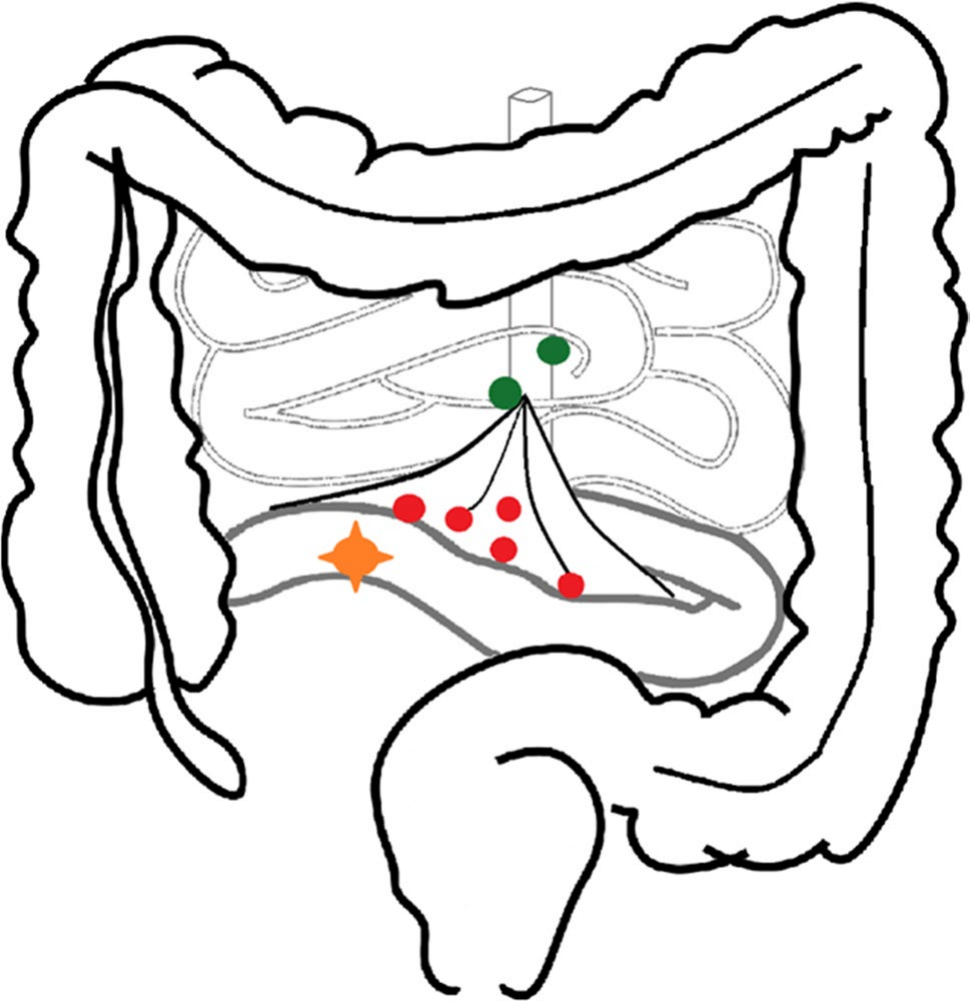
Mesenteric lymph node pattern. Orange: Primary tumor; red: locoregional lymph node metastases; green: aortocaval/central metastases; schematic for distant metastases not shown

Five groups of lymph node spread patterns were formed for 195 patients based on postoperative histopathological reports and preoperative staging imaging: Patients without LN metastases, locoregional, central (aortocaval), and other intraabdominal stations as well as their combination in regard to the classifications established by Lardière-Deguelte and colleagues (Fig. 1, Tables 1 and 4) [12]. For 13 patients, no histopathological report was available retrospectively.

Patients’ follow-up data was recorded until December 2020 at the Department of Hepatology and Gastroenterology of the Charité University Medicine in Berlin, or until death of a patient. Patients´ follow-up was performed according to ENETS consensus guidelines [25]. OS was defined as time between diagnosis and death or time of last visit (loss to follow-up). Progression-free survival (PFS) was calculated as time between diagnosis and first postoperative progress. Disease progression was defined through clinical assessment and cross-sectional imaging according to Response Evaluation Criteria in Solid Tumors (RECIST). Remission was defined as R0 resection without tumor detection in laboratory check-ups, radiological imaging and during clinical evaluation within the follow-up.

## Statistical analysis

The statistical analysis was performed using Statistical Package for the Social Science (SPSS) software version 27 (copyright IBM).

OS and PFS were calculated as primary endpoints of survival analysis using the Kaplan–Meier method and log-rank tests for univariate analysis of potentially prognostic factors. Analyzed prognostic factors were WHO classification subgroups, tumor stages, tumor grades, metastatic pattern, resection margins, LN ratio, and presence or absence of a carcinoid syndrome. Normal distributed data are presented as median and range. All *p* values < 0.05 were considered as statistically significant.

## Results

Between 2010 and 2020 a cohort of 208 patients with si-NET (90 male, 118 female, median age at diagnosis 62 (range 27–84 years)) were treated at the ENETS Centre of Excellence at the Charité University Medcine in Berlin. Median time of follow up was 60 months (range 0–254 months).

LN metastases were present in 174 (83.6%) patients, and in 34 (18.5%) patients multifocal si-NET was registered. A total of 132 (63.8%) patients presented with stage IV disease (UICC) at time of diagnosis. Metastases of the liver were the most common site of distant metastases and occurred in 113 (54.3%) patients at initial diagnosis (Table 1). Patients with liver metastases showed worse OS (*p* = 0.003) while patients with solely LN metastases were not (*p* = 0.198).

**Table 1:** Patients’ demographic and staging characteristics

Parameter	Patients	
Median age at diagnosis (years)	62 (27–84)	
Time to death/loss to follow up (months)	60 (0–254)	
Male gender	90 (43.3%)	
Status at diagnosis		
Carcinoid syndrome (*n* = 208)	Yes	77 (37.0%)
	No	117 (56.3%)
	Unknown	14 (6.7%)
Carcinoid heart disease (*n* = 208)	Yes	19 (9.1%)
	No	169 (81.3%)
	Unknown	20 (9.6%)
Liver metastases (*n* = 208)	Yes	113 (54.3%)
	No	92 (44.2%)
	Unknown	3 (1.5%)
Metachronous metastasis (*n* = 208)	Yes	57 (27.4%)
	No	147 (70.7%)
	Unknown	4 (1.9%)
UICC according to staging imaging	*n* = 207 missing *n* = 1	
0	4 (1.9%)	
I	8 (3.9%)	
II	10 (4.8%)	
III	53 (25.6%)	
IV	132 (63.8%)	
Number of affected Organ Systems Distant metastasis, excluding LN	*n* = 203 Missing *n* = 5	
0	71 (34.1%)	
1 + 2	116 (55.8%)	
≥ 3	16 (7.7%)	

*UICC* Union for International Cancer Control

Seventy-one (34.1%) patients presented without distant metastases and 21 (10.0%) patients with neither distant nor LN metastases. 116 (55.8%) patients had one or two metastasis-affected organ, 16 patients (7.7%) showed more than three affected organ systems. With liver and LN metastases being the most common sites, metastases of the peritoneum, bone metastases, and metasases of the ovaries were affected systems. In rare cases, patients presented with metasases of the mediastinum and chest cavity and cerebral metastases.

By measuring Ki67-positive cells/ MC in tumor specimens 113 (54.5%) si-NET were defined as G1 tumors, and 93 (44.7%) as G2 tumors, respectively (Table 2).

**Table 2:** Surgical and histopathological data

Parameter	Patients	
Surgical data	*n* = 183 Missing *n* = 25 without surgery	
Multifocal si-NET	Yes	34 (18.5%)
	No	124 (67.8%)
	Unknown	25 (13.7%)
Lymph node metastases, including staging imaging for patients without surgery	*n* = 195 Missing *n* = 13	
Locoregional (mesenteric root)	106 (51.0%)	
Regional + central	47 (22.6%)	
Central (aortocaval)	5 (2.4%)	
Local + distant (other abdominal stations, cervical, mediastinal)	16 (7.7%)	
No lymph node metastases	21 (10.1%)	
Histopathological data		
Grading, sampling from function of metastases or postoperative pathological report	*n* = 208	
G 1 G 2 G 3 G x	113 (54.5%)	
	93 (44.7%)	
	1 (0.5%)	
	1 (0.5%)	
Postoperative histology	*n* = 183	
T 0 T 1 T 2 T 3 T 4 T x	3 (1.6%)	
	13 (7.2%)	
	30 (16.4.0%)	
	78 (42.6%)	
	50 (27.3%)	
	9 (4.9%)	
N 0 N 1 N x	26 (14.6%)	
	143 (80.3%)	
	9 (5.1%)	
L 0 L 1 L x	48 (23.1%)	
	105 (50.5%)	
	30 (14.4%)	
V 0 V 1 V x	83 (45.4%)	
	63 (34.4%)	
	37 (20.2%)	
Pn 0 Pn 1 Pn x	13 (7.1%)	
	42 (23.0%)	
	128 (69.9)	
R 0 R 1 R 2 R x	125 (68.3%)	
	40 (21.9%)	
	6 (3.3%)	
	12 (6.5%)	

*T* Tumor infiltration; *G* grading; *N* lymph node metastases; *L* lymphangioinvasion; *V* angioinvasion; *Pn* perineuralinvasion; *R* residual tumor after 1st surgery

A total of 183 (87.9%) patients underwent surgery. Of those, 36 (19.6%) patients underwent re-resection to obtain R0 resection. Overall, 82 (39.4%) patients attained the status of complete remission in 1–3 surgeries. All operated patients with neither LN nor distant metastases (*n* = 21) remained remission-free during the observation period.

Indication for initial surgery was histologically confirmed si-NET or suspicion of si-NET based on radiological imaging and laboratory findings in 165 (90.1%) cases, while in 15 patients (8.2%) si-NET was diagnosed during an abdominal procedure for other reasons (hernia repair, appendectomy, gynecological procedures, etc.). In 40 of 183 (21.8%) operated patients, primary surgery was performed in an emergency setting (bowel obstruction, ischemia).

Intention of elective primary surgery was curative resection in 59 patients (32.2%) patients and palliative surgery as primary procedure in 47 (25.7%) patients. Of the palliative surgeries cytoreduction for support of other therapeutical approaches (i.e., alleviation of carcinoid symptoms) was performed in 19 patients (10.3%) (Table 3). Re-operation was performed in 72 (39.3%) patients. In 27 (37.5%) patients, re-operation was performed as secondary resection in the cause of incomplete first resection (R1) or when recurrence was confirmed and tumor was classified as resectable. In the cases of 56 (77.8%) patients, re-operation was performed due to early postoperative complications (such as insufficiency of anastomosis, intraabdominal abscess) and late postoperative complications (mainly adhesions and consecutive pain or ileus).

**Table 3:** Surgical parameters of the studied patients

	1st surgery	2nd surgery	3rd surgery
Total cohort	*n* = 183	*n* = 72 (39.3%)	*n* = 21 (11.4%)
Associated with si-NET	165 (90.1%)	56 (77.8%)	13 (59.1%)
Secondary resection	-	27 (37.5%)	9 (40.9%)
*Surgical indication*			
Elective curative resection	59 (32.2%)	17 (23.6%)	1 (4.5%)
Elective palliative resection	47 (25.7%)	8 (11.1%)	1 (4.5%)
Urgent/emergency	40 (21.8%)	15 (20.8%)	6 (28.5%)
Incidental finding	15 (8.2%)	-	-
Other/unknown	22 (12.0%)	11 (15.2%)	14 (66.6%)
Other si-NET associated	-	9 (12.5%)	-
*Bowel resection*	164 (89.6%)	51 (70.8%)	15 (71.4%)
Small bowel resection	60 (36.6%)	17 (33.3%)	5 (33.3%)
Ileocecal resection	21 (12.8%)	7 (13.7%)	1 (6.7%)
Right hemi colectomy	72 (43.9%)	11 (21.6%)	2 (13.3%)
Extended right hemi colectomy	4 (2.4%)	2 (3.9%)	-
Right hemi colectomy + small bowel resection	5 (3.0%)	-	-
Other	2 (1.2%)	14 (27.5%)	7 (46.7%)
*Liver resection*	48 (26.2%)	19 (26.4%)	3 (14.2%)
Sampling	18 (37.5%)	4 (21.1%)	1 (33.3%)
Atypical/enucleation	20 (41.7%)	11 (57.9%)	
Right hemi hepatectomy	7 (14.6%)	2 (10.5%)	
Left hemi hepatectomy	2 (4.2%)	-	
Other	1 (2.1%)	2 (10.5%)	2 (66.7%)
Resection of other organs	42 (22.9%)	28 (38.8%)	8 (38.0%)
Palliative Debulking	19 (10.3%)	12 (16.6%)	1 (4.7%)
Complete remission through surgery	57 (31.1%)	18 (25.0%)	7 (33.3%)

% of 2nd and 3rd surgery corresponds to total n of 1^st^ surgery. All other % correspond to the respective n of the column

Univariate analysis showed several significant negative prognostic factors for PFS, including Ki67-Index > 3% (*p* = 0.003) and Ki67 > 10% (*p* < 0.001), N + status (*p* = 0.003), distant metastases (*p* < 0.001), lymphatic- and vascular invasion (*p* = 0.020; *p* = 0.001), R1/R2 resection (*p* < 0.001), and number of affected organ systems (0 vs. 1 + 2) (*p* < 0.001) among others (Table 4). Multifocal si-NET showed no differences in PFS but had worse prognosis in OS (*p* = 0.007).

**Table 4:** Negative prognostic factors for progression free survival

Parameters at diagnosis	Yes	No	*P* value
Carcinoid syndrome (unknown = 14/208)	77 (39.7%)	117 (60.3%)	< 0.001
Hedinger syndrome (unknown = 20/208)	19 (10.1%)	169 (89.9%)	0.001
Hepatic metastases (unknown = 3/208)	113 (55.1%)	92 (44.9%)	< 0.001
Distant metastases (UICC 4) (unknown = 1/208)	132 (63.7%)	75 (36.3%)	< 0.001
Metachronous metastases (unknown 4/208)	57 (27.9%)	147 (72.1%)	< 0.001
N + status* N0 vs. N1 (unknown = 39/208)	143 (84.6%)	26 (15.4%)	0.003
Lymphovascular invasion (unknown 55/208)	105 (68.6%)	48 (31.4%)	0.020
Angioinvasion (unknown 62/208)	63 (43.2%)	83 (56.8%)	0.001
R + status* R0 vs. R1/ R2 (unknown 25/208)	125 (60.0%)	46 (22.1%)	< 0.001
Tumor infiltration T1 / T2 vs. T3 / T4 *n* = 171	T1 / T2	T3 / T4	< 0.001
	43 (25.1%)	128 (74.9%)	
Ki67 1–2% vs. ≥ 3% (unknown 12/208)	Ki67 1 – 2%	Ki67 ≥ 3%	0.003
	108 (51.9%)	88 (42.3%)	
Ki67 1–9% vs. 10–20% (unknown 12/208)	Ki67 1 – 9%	Ki67 10 – 20%	< 0.001
	170 (81.7%)	26 (12.5%)	
Ki67 3–9% vs. 10–20% *n* = 74/208	Ki67 3 – 9%	Ki67 10 – 20%	0.004
	62 (29.8%)	26 (12.5%)	
Afflicted organs at diagnosis (0 vs. 1 + 2) *n* = 187/208	0	1 + 2	0.001
	71 (34.1%)	116 (55.8%)	
Afflicted organs at diagnosis (0 vs. ≥ 3) *n* = 181/208	0	≥ 3	< 0.001
	71 (34.1%)	16 (7.7%)	

*N* + Lymph node metastases; *postoperative histology; *R* + presence of residual tumor after surgery; *T* tumor infiltration; *UICC* Union for International Cancer Control

**Fig. 2:**
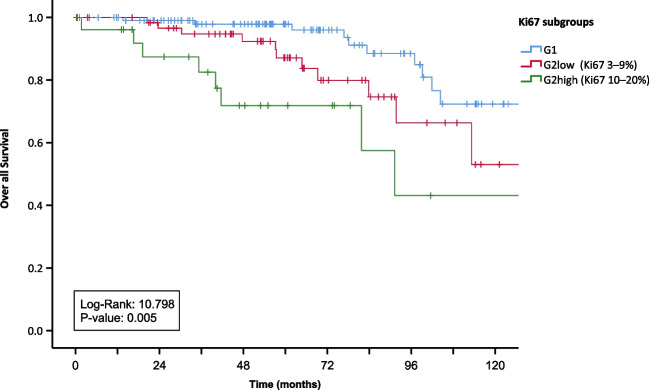
Kaplan–Meier curves of overall survival for the G1 group and the G2 group split in G2 low (Ki67 3–9%) and G2 high (Ki67 10–20%)

**Fig. 3:**
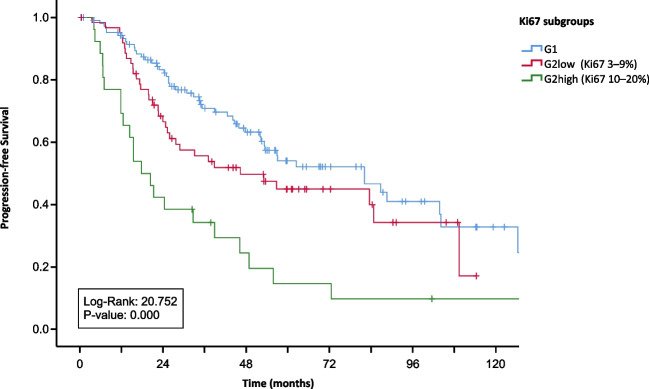
Kaplan–Meier curves of progression-free survival for the G1 group and the G2 group split in G2 low (Ki67 3–9%) and G2 high (Ki67 10–20%)

Patients with G2 tumors (Ki67 3–20%) displayed worse OS compared to patients with G1 tumors (*p* = 0.005) and showed a significantly earlier progression. When splitting the G2 group in two subgroups (G2 low: Ki67: 3–9% and G2 high: Ki67: 10–20%), they displayed significant differences in OS (*p* = 0.005) (Fig. 2) and PFS (*p* < 0.001) (Fig. 2 + 3) compared to G1 tumors. (Fig. 3)

Among 176 patients with full follow-up data, 50 of 106 patients (47.2%) in the G1-group experienced complete remission after surgery. This was the case for 19 of 47 patients (40.4%) in the low-G2-group (Ki67 3–9%) and only for 2 of 23 patients (8.7%) in the high-G2-group (Ki67 10–20%).

**Fig. 4:**
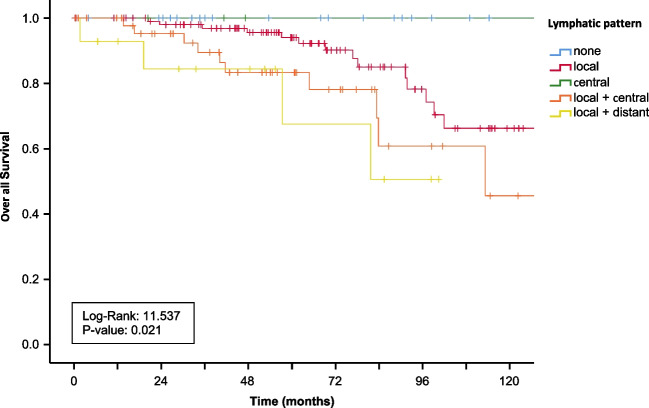
Kaplan–Meier curves of overall survival of all patients grouped for mesenterial lymph node pattern

Addressing the pattern of metastases, most patients had locoregional LN metastases in the mesenteric root (*n* = 153), while only few patients presented with only aortocaval/central LN metastases (*n* = 4) (Table 5). All patients with distant LN metastases showed regional LN metastases as well. Patients with isolated locoregional LN metastases (*n* = 106) had a significantly longer PFS (*p* = 0.012) as well as OS (*p* = 0.044) when compared with other patterns of metastases (Fig. 4 + 5).

**Table 5:** Lymph node pattern of the patient cohort related to grading and *T* status

		Grading (*n* = 195, *n* = 13 missing)			*T* status (*n* = 168, *n* = 40 missing)			
*Lymph node pattern*	Total cohort (*n* = 208)	G1 (*n* = 112)	G2 Ki67 3–9% (*n* = 55)	G2 Ki67 10–20% (*n* = 28)	T1 (*n* = 13)	T2 (*n* = 29)	T3 (*n* = 76)	T4 (*n* = 50)
Locoregional (mesenteric root)	106 (51.0%)	65 (58.0%)	30 (54.5%)	11 (39.3%)	5 (38.4%)	19 (65.5%)	44 (55.7%)	27 (54.0%)
Regional + central	47 (22.6%)	22 (19.7%)	15 (27.3%)	10 (35.7%)	0	3 (10.3%)	19 (24.0%)	18 (36.0%)
Central (aortocaval)	5 (2.4%)	3 (2.7%)	1 (1.8%)	1 (3.5%)	1 (7.7%)	1 (3.5%)	3 (3.7%)	0
Local + distant (other abdominal stations, cervical, mediastinal)	16 (7.7%)	8 (7.1%)	3 (5.5%)	5 (17.9%)	0	0	5 (6.3%)	5 (10.0%)
No lymph node metastases	21 (10.1%)	14 (12.5%)	6 (10.9%)	1 (3.5%)	7 (53.9%)	6 (20.7%)	5 (6.3%)	0
Missing data	13 (6.2%)							

*G* Grading; *T* tumor infiltration

None of the patients who solely displayed central metastases died during the follow-up period, neither did patients without LN metastases. Patients with distant LN metastases presented with poorer OS and PFS (*p* = 0.003). Patients with either locoregional or aortocaval/central disease had better outcome in both endpoints in comparison with patients with locoregional and central metastases (Fig. 4 + 5).

**Fig. 5:**
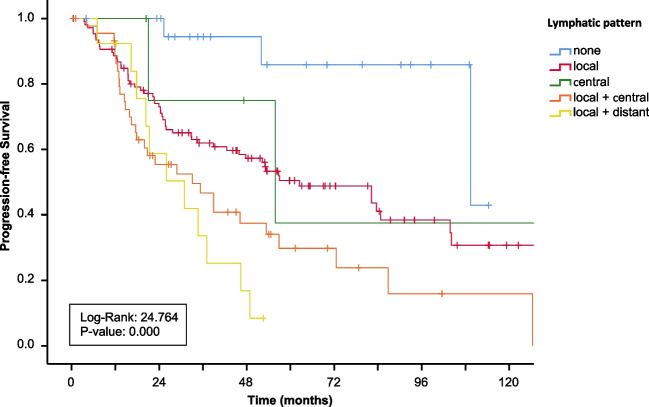
Kaplan–Meier curves of progression-free survival of all patients grouped for mesenterial lymph node pattern

Tumor grading correlated with the pattern of LN metastases. Of 21 (10.1%) patients without LN metastases, 14 (66.7%) had a G1 tumor. Locoregionally limited disease presented mainly in patients with Ki67 < 10% (89.6%) (Fig. 5). Tumor infiltration level (T status) displayed correlation with the LN pattern as well. Of 40 (19.2%) patients with local and central manifestation 37 (92.5%) fell onto T3 and T4 tumors. Distant LN metastases (*n* = 10) were only seen with T3 and T4 tumors. None of the 13 (6.3%) patients with T1 tumors displayed distant LN metastases at all (Table 5).

## Discussion

The heterogeneity of NETs and their differences in prognosis has been documented in many studies, depicting localization, functionality, patient age, and TNM stage as important factors for prognosis. For NETs the grading system, by measuring the ratio of Ki67 positive cells, is of utmost importance, nowadays, when patient prognosis is addressed [26, 27].

In this study, proliferation index Ki67 was confirmed as the most important independent marker for OS and PFS. Addressing further histopathological assessment lymphovascular- (L) and venous (V) invasion were shown as negative prognostic factors for PFS, but not for OS. Statistical analysis for perineural invasion (Pn) was not feasible as it had not been evaluated in the majority of histological specimens. Interestingly, none of the patients with Pn 0 status (*n* = 12) died during observation. L, V, and Pn are established factors in colorectal carcinoma but their significance for si-NET is still under discussion [28].

The role of grading (Ki67 index) as a prognostic factor for GEP-NET was validated in several studies [14, 20, 26, 27]. The distribution of G1 and G2 tumors among our patient cohort was nearly balanced with 54.5% G1 tumors and 44.7% G2 tumors. Our study confirms earlier findings that patients with G2 tumors display worse OS compared to patients with G1 tumors and show a significantly earlier progression postoperatively (*p* = 0.003) [14, 29].

The two G2 subgroups: G2 low (Ki67 3–9%) and G2 high (Ki67 10–20%) displayed significant differences in OS and PFS (Figs. 2 and 3). Using these G2 subgroups, complete remission through surgery was less often achieved in patients with higher Ki67 index (> 10%). Only 8.7% of patients in the G2-high-group stayed remission-free during observation period.

Discussion about Ki67 cut-off values for GEP-NET is ongoing. Special attention is paid to the transition between G1 and G2 tumors, mostly in pancreatic tumors (pNET) [18, 27, 29]. Still only few studies discuss the role of grading for ileal NET [19, 20, 29, 30].

In a retrospective study Panzuto et al. [29] could show worse OS for Ki67 > 5% when used as cut-off value between G1 and G2 but not for PFS in advanced si-NET. Sun et al. [19] could show Ki67 > 5% to be a negative prognostic factor for patients with stage IV disease, but not in stages I–III. We did not investigate cut-off between G1 and G2. In our cohort, however, patients with a Ki67 > 10% displayed significantly worse progression-free survival compared to patients with Ki67 ≤ 9% (*p* < 0.001). Ki67 > 10% as predictor for shorter PFS in si-NET was also shown in the Study of Lanreotide Autogel in Non-functioning Entero-pancreatic Endocrine Tumors (CLARINET FORTE) study [31].

These findings show the imbalance and heterogeneity of si-NET, especially in G2 group. Therefore, investigating only the difference between G1 and G2 tumors might not be sufficient for si-NET and differences within the G2 group should be considered as well.

In agreement with the results from Cavalcanti et al. [20], we acknowledge the possibility of biological differences in site of tumor origin in midgut NET (pNET vs. si-NET). Consequently, site-specific grading within the GEP-NET group should be evaluated.

Due to mostly small primaries and relatively slow progression si-NET are rarely diagnosed before metastases have occurred [32], Si-NET has been associated with mesenteric lesions even in the smallest tumors and metastases often grow larger than the primary leading to mesenteric fibrosis, ischemia, or bowel obstruction [8, 10, 33]. N + status was confirmed as negative prognostic factor for postoperative PFS (*p* = 0.003). This underlines that adequate lymphadenectomy during resection of primary tumors is crucial in surgical attempt of R0 resection. Significance of mesenterial LN metastases is widely discussed in the literature [12, 34–36].

In contrast to other studies, we could neither confirm the number of positive resected LN nor the LN ratio as independent prognostic factor for OS and PFS [35–37]. That the number of resected LNs does not parallel an improved outcome is not surprising to us, since the number of affected and resected LNs increases with disease extension. The limited number of patients in our study as well as the lack of standardization in operative procedures may explain the lack of prognostic importance of the LN ratio in our cohort.

Resection of small bowel and affected lymph nodes in si-NET patients is limited to prevent postoperative short bowel syndrome and length of resected small intestine does not always correlate with number of resected LNs [12]. The benefit of an extended lymph node dissection is not indicated by our data. However, the importance of extended LN resection in si-NET has been shown by several other publications. Landry et al. included more than 1300 patients who underwent surgery. They found that patients with > 7 LN removed experienced better cancer specific survival than < 7 LN [34]. This is also reflected in current guidelines [8].

N + status without other organ metastasis is classified as stage III disease, but not all patterns present with the same outcome. In attempt of further surgical treatment options, Öhrvall et al. introduced intraoperative mapping of mesenteric metastases in si-NET [10]. Metastases were categorized in four stages of midgut carcinoid metastases. A similar approach was taken by Lardière-Deguelte et al. proposing a preoperative morphological classification in five stages [12]. Following the idea of LN mapping for our study cohort, LN patterns were divided as formerly described. Patients with isolated locoregional disease showed better PFS and OS in comparison to other groups. Interestingly, none of 4 patients presenting without regional but with singular central metastasis died during follow-up if an R0-resection was achieved. It is not known why in some GEP-NET lymphatic metastases seem to skip regional station and form central LN metastases. In our cohort patients with distant LN metastases presented with poorer PFS (*p* = 0.003), suggesting that distant intraabdominal LN metastases should be considered as separate LN entity. To our knowledge, this is the first time that this correlation has been described as an independent prognostic factor for OS and PFS.

Stages I–III si-NET have an excellent prognosis if R0 resection is attained [8, 15, 30]. Postoperative remaining metastases (R +) are associated with poor OS and remission free survival [14, 38]. In our analysis, there was no significant difference between the R1 and R2 group.

Controversial results concerning resection of primary, locoregional metastases and distant metastases in si-NET have been published and discussed in various ways [30, 39, 40]. Debates considering resection margins are ongoing [12, 41,] and intraoperative lymphatic mapping has been suggested to be of help to limit the extent of small bowel resection [8, 41].

ENETS guidelines recommend surgery following the principle of oncological resection in the small intestine. If the primary is located in the terminal ileum, right hemicolectomy is mostly required [8, 33, 40]. A standard protocol for the surgical approach is lacking, especially for NET located in the proximal part of the terminal ileum. As recently described by Evers et al. [15], conventional right hemicolectomy is performed for stages I–III NET localized in the distal 40 cm of the ileum. This was associated with reduced risk of disease recurrence. Our data did not confirm right-sided hemicolectomy as prognostic marker. These results might be related to the fact that we included all tumor stages I–IV and patients with curative and palliative resections.

In symptomatic patients due to intestinal obstruction, ischemia or angulation, resection of tumor can be inevitable to avert further deterioration [8, 42]. In patients with stage IV disease, resection of the primary has been advocated even in presence of unresectable liver metastases [30, 42, 43]. In our study, 106 of 132 patients with stage IV disease underwent surgery and 8 (7.5%) demonstrated complete remission during the observation period. All of these patients had isolated hepatic metastases.

In 98 patients, surgical resection was included into the interdisciplinary therapeutic modalities of stage IV si-NET, even in palliative intention.

As already established in previous studies, gender did not influence OS and PFS in si-NET [15, 30, 44].

Further, understanding of the biology of primary and its metastases might shed light on why metastatic spread pattern in the mesenteric root varies between patients. Scarpa et al. investigated the molecular landscape of pNET and si-NET. While a significant number of alterations was found in pNET the same holds not true for si-NET (e.g., no recurrent driver event and mutation rate 3 times lower than in pNET). Overall, the molecular pattern of si-NET and its metastases seems to be more defined by gene methylations then by mutation [45]. Understanding molecular pathways and connecting those to clinical findings is crucial for future targeted therapies.

Furthermore, a recent study by Mäkinen et al. indicates a high genomic diversity in multifocal si-NET suggesting the tumors to develop independently. Further, they describe that multiple metastases in the same patient can originate from any of the primary tumors, underlining the importance of a proper manual screening for multifocal NET [46].

Given the scarceness of si-NET, this study shows a large population of 208 patients. The study is limited in its retrospective nature, partially incomplete data, and potential selection bias of a single-center observation.

However, the single-center design ensured a relatively homogeneous multidisciplinary management at a tertiary treatment center with access to all available treatment modalities as well as an experienced surgical team specialized in endocrine and visceral surgery. Randomized controlled prospective studies are needed to generate and understand criteria for si-NET treatment and surgical approaches. Due to the rarity and the often slow and indolent course of si-NET such trials, however, are difficult to establish.

## Conclusion

In summary, we herein present real-life data in a large cohort of patients regarding the prognosis of si-NET. While known factors for OS and remission free survival in si-NET patients were confirmed, we identified LN pattern and discrimination of the moderately differentiated G2-group as new prognostic factors. Patients with G2 tumors with higher Ki67 index > 10% had significantly worse overall and remission free survival than patients with G2 tumors graded between 3 and 9%. Both results might impact adjuvant treatment and surgical strategy of si-NET patients.
